# Regional divergence and time trends in the prevalence of gestational diabetes mellitus: a national Danish cohort study

**DOI:** 10.1007/s00592-022-02013-8

**Published:** 2022-12-20

**Authors:** Cathrine M. Scheuer, Maria H. Andersen, Elisabeth R. Mathiesen, Lene Ringholm, Clara L. Müller, Jun-Mei Truong, Michelle M. Lie-Olesen, Martin Overgaard, H. David McIntyre, Dorte M. Jensen, Peter Damm, Tine D. Clausen

**Affiliations:** 1 Department of Gynaecology and Obstetrics, Nordsjællands Hospital, Hillerød, Denmark; 2 Department of Endocrinology and Metabolism, Center for Pregnant Women with Diabetes, Copenhagen, Denmark; 3 Department of Clinical Medicine, University of Copenhagen, Copenhagen, Denmark; 4 Department of Clinical Biochemistry, Odense University Hospital, Odense, Denmark; 5 Mater Clinical Unit, Faculty of Medicine, The University of Queensland, Brisbane, Australia; 6 Steno Diabetes Center Odense, Odense University Hospital, Odense, Denmark; 7 Department of Gynecology and Obstetrics, Odense University Hospital, Odense, Denmark; 8 Department of Clinical Research, Faculty of Health Sciences, University of Southern Denmark, Odense, Denmark; 9 Department of Obstetrics, Center for Pregnant Women with Diabetes, Rigshospitalet, Copenhagen, Denmark

**Keywords:** Gestational diabetes mellitus, Denmark, Prevalence, Pregnancy, Registries

## Abstract

**Aims:**

To evaluate the prevalence and time trends of gestational diabetes mellitus (GDM) across the five regions of Denmark with uniform national guidelines for screening and diagnosing GDM.

**Methods:**

This register-based national cohort study included 287,684 births from 2013 to 2017. Trends in GDM prevalence over time and differences between the five regions were evaluated. Crude and adjusted odd ratios (ORs) for GDM were calculated including potential confounding clinical risk factors as age, BMI, educational level, marital status, parity, country of origin and assisted reproduction.

**Results:**

From 2013 to 2017, GDM prevalence in Denmark increased by 7% per year (OR 1.07, 95% CI 1.06–1.09, *P* < 0.001). GDM prevalence varied considerably between regions and ranged from 3.0 to 5.9% in 2017, corresponding to a maximal regional difference of 97%. In crude analyses, the risk of GDM in 2017 was significantly different in four of five regions compared to the remaining regions (OR ranging from 0.60 to 1.55), and these differences persisted after adjusting for confounding clinical risk factors (adjusted OR: 0.59–1.45).

**Conclusion:**

The prevalence of GDM increased over time in all Danish regions with substantial regional divergence. Up to a 97%, difference in GDM prevalence was observed between Danish regions, which was not explained by available clinical risk factors. This occurred despite national guidelines and raises the question of whether regional variations in screening efficacy, diagnostic procedures or inequality in clinical health care access may explain the observed differences.

**Supplementary Information:**

The online version contains supplementary material available at 10.1007/s00592-022-02013-8.

## Introduction

Gestational diabetes mellitus (GDM) is a common medical condition complicating pregnancy. Nevertheless, its prevalence varies markedly in the international literature, presumably due to differences in screening methods, diagnostic criteria and the background population’s risk of diabetes [1–3].

Scandinavia, including Denmark, has a long tradition of risk factor-based screening for GDM [4]. When the present Danish risk factor-based screening procedure was first developed 20 years ago, 36% of the pregnant women had pre-pregnancy risk factors for GDM [5]. A recent assessment from Nordsjællands Hospital in Hillerød, Denmark, found that 49% of pregnant women completed an oral glucose tolerance test (OGTT) (unpublished data); however, the proportion of women with pre-pregnancy risk factors for GDM in the Danish population at present is unknown. Whether risk factor-based screening detects fewer women with GDM compared to universal screening is debated [6], yet, approximately 20% of GDM cases are undoubtedly missed by the current Danish screening strategy [5].

Previous Danish studies on GDM prevalence have focused on national data, but to our knowledge, no studies have evaluated regional differences in GDM prevalence. Such differences may occur due to differences in risk factor profiles in subpopulations, the efficacy of the risk factor identification process, pre-analytic and analytic factors in association with the diagnostic test and accuracy of diagnostic coding. All these factors could be reflected in the national registries as regional differences in GDM prevalence.

In the present study, we aimed to describe the prevalence of GDM and its change over time nationally and within the five Danish regions and investigate whether clinical risk factors could explain any observed regional differences.

## Methods

### Data sources and study population

This was a population-based register study including 287,684 live and stillbirths in Denmark from 2013 to 2017 at gestational age 22–43 weeks. For infants, only data on singleton pregnancies were reported (*N* = 286,642). Data were drawn from the National Patient Register, the Medical Birth Register and the National Prescription Registry. The study was approved by the Danish Data Protection Agency at the Capital Region (NOH-2015-016) and did not, by study design, include informed consent from participants. Detailed description of variable classification can be found in Online Resource 1.

### Main outcome measure

The main outcome was the diagnosis of GDM. We identified women diagnosed with GDM if they were registered with an ICD-10 code for GDM (D0244, D0244B, D0244C, D0244D or D0244E) within 180 days prior to or 30 days after birth.

The Danish screening indications for GDM included the presence of at least one of the following six pre-pregnancy risk factors: GDM in a previous pregnancy, prior birth of a macrosomic infant (≥ 4500 g), pre-pregnancy body mass index (BMI) ≥ 27 kg/m^2^, first-degree family history of diabetes, polycystic ovarian syndrome and multiple pregnancies. Women presenting one or more of these risk factors were offered a diagnostic test for GDM at 24–28 weeks. Women with GDM in a previous pregnancy or at least two other risk factors were additionally offered early testing (10–20 weeks). Furthermore, glucosuria detected any time during pregnancy (≥ 5.5 mmol/L or 1 +) elicited a diagnostic test unless a normal test was completed within four weeks.

GDM was diagnosed by a 2 h venous plasma (or capillary whole blood) glucose value ≥ 9.0 mmol/L during a 75 g OGTT.

### Independent variable

The primary independent variable was the Danish administrative region in which the birth took place. Categorization of the regions adhered to the national division of Denmark into five regions: the Capital Region, the Zealand Region, the Southern Region, the Central Region and the Northern Region. In sensitivity analyses, the prevalence of GDM was further explored for the 21 individual Danish obstetric departments.

### Potential confounding clinical risk factors for GDM

Potential confounding clinical risk factors for GDM available from the Danish registers included maternal age at delivery (years), pre-pregnancy BMI (kg/m^2^), educational level (< 12 versus ≥ 12 years’ schooling), marital status (married vs non-married), parity (nulliparous vs multiparous), country of origin (Danish vs Non-Danish) and use of assisted reproduction (yes vs no).

### Statistical analyses

Categorical variables were reported as percentage (*N*) and continuous variables as mean (standard deviation) for data following the normal distribution or median (2.5–97.5 percentile) when not. A generalized binary logistic regression model with GDM as dependent variable (yes vs no) and birth year as covariate was used to evaluate the trend in GDM prevalence in Denmark from 2013 to 2017. Further, the trend in GDM prevalence in the individual regions was explored in a model including the main effects of “year” and “region” in addition to an interaction term “year*region.”

We examined associations between the individual regions, potential confounding clinical risk factors available from the Danish registers and GDM using odds ratios (ORs) and corresponding 95% confidence intervals (CI) calculated from logistic regression models using the remaining four regions as reference. Crude ORs were estimated in univariate analyses with region as independent and GDM as dependent variable. Adjusted ORs of GDM were estimated in stepwise multivariate analyses adjusting for maternal age (model 1), adding pre-pregnancy BMI to model 1 (model 2) and finally adding educational level, marital status, parity, country of origin and assisted reproduction (model 3). Models were not reduced.

Risk, prevalence and clinical risk factors for GDM were explored in sensitivity analyses for each of the 21 Danish obstetric departments in 2017.

The significance level was set at 5% or less (*P* ≤ 0.05), and we used complete case analyses reporting *p* values as two-sided assuming non-equal variances. We did not adjust for multiple testing. All analyses were performed using IBM SPSS Statistics, version 27.0.

## Results

In 2017, the prevalence of GDM varied considerably between regions. The overall prevalence of GDM in Denmark was 4.2% but ranged from 3.0 in the Capital Region to 5.9% in the Southern Region corresponding to a difference of 97% (Table 1).

**Table 1:** GDM prevalence and clinical risk factors in the Danish regions, 2017

	Number of births (% and *N*)	GDM prevalence (% and *N*)	Age at delivery (years)	Pre-pregnancy BMI (kg/m^2^)^a^	High educational level	Married	Nulliparous (no prior births)	Non-Danish country of origin	Assisted reproduction
Denmark (all regions)	100% (60,006)	4.2% (2506)	30.3 (± 5.0)	23.3 (18.0–38.1)	24.8% (14,594)	45.8% (27,433)	49.2% (29,475)	22.5% (13,460)	9.2% (5495)
Capital Region	36.7% (22,019)	3.0% (653)	31.3 (± 4.9)	22.6 (17.9–36.1)	37.8% (8130)	48.1% (10,560)	53.0% (11,658)	28.1% (6183)	10.1% (2217)
Zealand Region	10.9% (6546)	5.1% (332)	29.4 (± 5.2)	24.1 (17.9–39.7)	12.7% (815)	42.6% (2786)	46.8% (3063)	20.6% (1349)	6.6% (429)
Southern Region	13.8% (8304)	5.9% (491)	29.6 (± 4.9)	24.0 (18.1–40.0)	15.1% (1229)	43.2% (3577)	44.5% (3693)	20.3% (1682)	7.5% (626)
Central Region	29.5% (17,713)	4.2% (738)	30.0 (± 4.8)	23.4 (18.0–32.2)	20.4% (3550)	46.2% (8176)	46.0% (8118)	19.2% (3390)	10.0% (1772)
Northern Region	9.0% (5424)	5.4% (292)	29.6 (± 4.9)	24.0 (17.9–39.4)	16.4% (870)	43.1% (2334)	54.3% (2943)	15.8% (856)	8.3% (451)

GDM: Gestational diabetes mellitus. BMI: Body mass index

Categorical data are given as % (*N*) and continuous as mean (± SD) or ^a^median (2.5–97.5 percentiles) if data were not normally distributed. Data from multiple pregnancies are excluded from the neonatal outcomes (*N* = 1042). Missing data: pre-pregnancy BMI *N* = 1551; educational level *N* = 1185; marital status *N* = 103; parity *N* = 71; country of origin *N* = 95; birth weight *N* = 388

**Table 2:** Crude and adjusted odds ratios for GDM in each of the five Danish regions in 2017 compared to the remaining four regions

	Crude odds ratios		Adjusted odds ratios					
			Model 1^a^ *N* = 60,006		Model 2^b^ *N* = 58,455		Model 3^c^ *N* = 57,281	
	OR	95% CI	OR	95% CI	OR	95% CI	OR	95% CI
Capital Region	**0.60**	**0.54–0.65**	**0.53**	**0.49–0.58**	**0.64**	**0.58–0.70**	**0.59**	**0.54–0.65**
Zealand Region	**1.26**	**1.12–1.42**	**1.34**	**1.19–1.51**	**1.16**	**1.02–1.31**	**1.17**	**1.03–1.33**
Southern Region	**1.55**	**1.40–1.72**	**1.64**	**1.48–1.82**	**1.43**	**1.29–1.59**	**1.45**	**1.30–1.62**
Central Region	1.00	0.91–1.09	1.03	0.95–1.13	1.02	0.93–1.11	1.05	0.96–1.15
Northern Region	**1.35**	**1.19–1.53**	**1.43**	**1.26–1.62**	**1.30**	**1.15–1.48**	**1.32**	**1.15–1.50**

GDM: Gestational diabetes mellitus. OR: Odds ratio. CI: Confidence interval.

ORs marked bold are significant (*P* ≤ 0.05).

^a^Model 1 adjusted for age at delivery.

^b^Model 2 adjusted for model 1 and pre-pregnancy BMI.

^c^Model 3 adjusted for model 2 and educational level, marital status, parity, country of origin and assisted reproduction

### Trend in GDM prevalence from 2013 to 2017

**Fig. 1:**
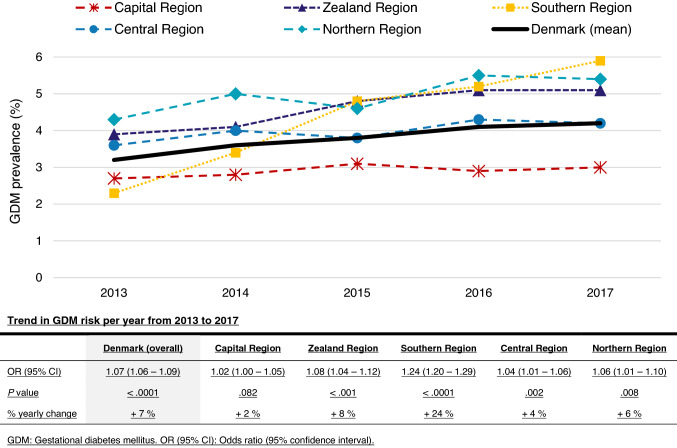
Regional GDM prevalence in Denmark from 2013 to 2017

From 2013 to 2017, GDM prevalence in Denmark increased significantly by 7% per year (OR 1.07, 95% CI 1.06–1.09, *P* < 0.001), with a notably steep increase in the Southern Region from 2.3 to 5.9% (Fig. 1). In each region, this trend was significant except for the Capital Region, which was also the region with the lowest GDM prevalence (Fig. 1). There was a significant difference in the time trends of GDM between regions (*P* < 0.0001) (data not shown); however, when excluding the Southern Region from the analysis, the interaction was no longer significant (*P* = 0.131) (data not shown).

### Regional variations in prevalence, clinical risk factors and risk

The distribution of potential confounding clinical risk factors did not appear uniform across regions, in particular with variations in the country of origin and educational level: Women with a non-Danish country of origin were most frequent in the Capital Region (28.1%) and least in the Northern (15.8%), while having a high educational level was most prevalent among women in the Capital Region (37.8%) compared to a range from 12.7 to 20.4% in the other four regions. By contrast, neonatal characteristics (infant sex, birth weight and GA at birth) did not differ between regions (data not shown).

The risk of GDM was significantly different in four of five regions compared to the remaining regions (Table 2). Estimates were essentially similar in the fully adjusted multivariate model (model 3), which demonstrated the highest OR in the Southern Region (OR 1.45, 95% CI 1.30–1.62), followed by the Northern Region (OR 1.32, 95% CI 1.15–1.50) and the Zealand Region (OR 1.17, 95% CI 1.03–1.33) (model 3). As the only region, the Central Region did not differ from the remaining regions (OR 1.05, 95% CI 0.96–1.15), whereas the Capital Region was the sole region demonstrating a lower risk of GDM (OR 0.59, 95% CI 0.54–0.65) (model 3).

In sensitivity analyses, the frequency of the majority of the clinical risk factors was significantly different for each obstetric department both within each region and compared to the remaining Danish departments, and the prevalence of GDM was similarly different from the national mean for most departments (Online Resource 2). Variations in OR of GDM were observed within regions. For example, in the Zealand Region, considering Hillerød as reference, the highest OR for GDM was seen in Roskilde (OR 2.13, 95% CI 1.67–2.71) and the lowest in Nykøbing Falster (OR 0.19, 95% CI 0.08–0.47) (Online Resource 3, model 3).

## Discussion

In this nationwide register-based cohort study of women giving birth in Denmark from 2013 to 2017, the prevalence of GDM increased by 7% per year. We observed a difference in the prevalence of GDM of up to 97% between regions despite uniform national guidelines for screening and diagnosing GDM. These differences could not be explained by regional differences in well-known clinical risk factors for GDM. Similarly, the observed distinct steep increase in the Southern Region was unexplained by any known change in population characteristics. Furthermore, we found substantial differences in GDM prevalence between the individual obstetric departments within each region, which again were not explained by dissimilarities in clinical risk factors.

### Existing literature on diverging GDM prevalence

The prevalence of GDM varies considerably worldwide and even within European countries. The variation is partly due to heterogeneity in screening procedures, diagnostics thresholds and population risk profiles, which makes comparison across countries challenging [1–3]. Despite the current focus on this topic, local within-country differences in GDM prevalence are less commonly evaluated, and results are not consistent. Aydın and colleagues found evenly distributed GDM prevalence between 12 regions of Turkey, even when comparing rural and urban areas [7]. Conversely, in a large cross-sectional study including pregnant women from all states of India, Swaminathan and colleagues describe marked variations in GDM prevalence across states, and even though some of the determining factors (i.e., maternal age > 35 years, BMI ≥ 27.5 kg/m^2^ and increasing wealth) were associated with GDM, none of these covariates varied significantly across states [8]. Correspondingly, an Australian review from 2020 highlighted the profound challenges of geographical inequality in health advantages and access to medical care that compound the risk profile in a time of increasing GDM burden, with an appeal for local adaptations in the management of women at risk [9].

### Diverging and increasing GDM prevalence from the Danish perspective

No prior studies have evaluated the regional distribution of GDM prevalence in Denmark or the underlying clinical or social differences. Though certain clinical, demographic and socioeconomic characteristics—in particular BMI and ethnicity—are associated with the risk of GDM [10–12], adjusting for these factors did not change the regional difference in GDM risk in our study.

In Denmark, the screening procedure, diagnostic test and threshold follow standardized national guidelines. However, these guidelines deal solely with indications for and timing of screening, whereas there is no uniform agreement regarding the laboratory procedures of the OGTT. Thus, such important procedural factors influencing the GDM prevalence are not necessarily similar between regions. It is well known that many components can influence the results of an OGTT and that the diagnosis of GDM relies on several pre-analytical, analytical and post-analytical factors with potential resulting bias in glucose measurements, which potentially contribute to differences in the final glucose value and subsequently the prevalence of GDM [13, 14].

Previous studies have demonstrated that glucose measurements by capillary whole blood result in low diagnostic accuracy and significantly lower glucose levels than measurements by venous plasma [15, 16]. In Denmark, there is no uniform agreement regarding the use of venous or capillary samples and this variance may in part explain the observed regional differences. In fact, the highest increase in prevalence in the Southern Region from 2013 to 2017 coincided with a shift in method in Odense from HemoCue measuring capillary blood to Abbott Architect measuring venous plasma. Another considerable but often neglected determinant for the OGTT-derived GDM diagnosis is the information given to the woman prior to testing [13], e.g., the duration of fasting, is she advised to eat a specific diet and not to exercise prior to testing and can she complete the test despite being treated with antibiotics? Further, variations in the percentage of non-attendees to the offered diagnostic test among women with pre-pregnancy risk factors could result in different numbers of detected cases, as we observed throughout the regions of Denmark. These factors could potentially influence the prevalence markedly if performed differently between regions, but no previous studies have compared the GDM screening procedure between Danish regions.

In addition, the consistency of diagnosis registration to the National databases will influence the registered disease prevalence. Previous studies have found inconsistency in the reporting of GDM prevalence between data sources [17–20]. For example, Lawrence and colleagues reported a proportion of agreement of only 0.71 (95% CI 0.66–0.76) for the registration of GDM diagnosis between two different clinical databases and laboratory glucose measurements [19]. Information on diagnosis coding was not available for validation analyses in our study but could—based on the literature—be a contributing reason for the regional differences in prevalence.

Finally, inequality in access to clinical health care could be a contributing cause of regional differences, as discussed in the Australian review from 2020 [9]. Such variation may explain why we observed the lowest GDM prevalence among women in Nykøbing Falster, who, despite this low rate of GDM, had the highest BMI and lowest educational level compared to the other Danish obstetric departments.

In addition to the abovementioned potential confounders that are unavailable from the Danish registries, unmeasured confounding caused by socioeconomic factors would presumably have contributed to our findings.

In accordance with the existing literature, we observed an increase in the prevalence of GDM over time [21]. Considering the worldwide increase in obesity, the most prominent risk factor of GDM, one could reasonably expect increased BMI to partly explain a simultaneous rise in GDM prevalence [22–24], and such increase did occur in our study population from 2013 to 2017 [25]. A rising GDM prevalence could also be caused by changes in a palette of procedural or administrative factors, including better identification of women at risk by the general practitioner, a higher attendance rate for GDM testing among women at risk, perhaps due to better patient information, or a shift from capillary whole blood to venous plasma.

The significant difference in trends between regions was driven by the steep increase in the South Region, since the difference did not persist when excluding the South Region from the analysis. In contrast, the Capital Region was the sole region without a significant increase in GDM risk over time, which could be partly explained by a more favorable distribution of clinical risk factors among women giving birth in the Capital Region. However, in both cases, adjusting for the available clinical risk factors did not change the difference in GDM risk between regions.

Altogether, these observations regarding factors influencing the prevalence of GDM and our observations of regional differences in prevalence support the hypothesis that local structural differences in diagnostic procedures may affect the number of women tested for and identified with GDM. Insights into such determinants of disease prevalence are valuable in future strategies for GDM monitoring. Understanding the impact of such determinants could be clarified by future research investigating to which extent the national guidelines for screening and diagnosing GDM are met by the individual departments. Further, evaluations of whether the OGTT procedures and glucose analyses throughout the Danish hospital laboratories follow a standardized practice would add valuable information on possible explanatory factors.

### Strengths and limitations

The main strength of our study is the large sample size and few missing values, which provide a unique opportunity to evaluate both national and local differences in demographics and potential confounding clinical risk factors for GDM. The study also has some limitations, primarily due to the register-based design. Despite inclusion of several known confounders for GDM, it is a limitation that we (apart from pre-pregnancy BMI) did not have access to the other pre-pregnancy risk factors, which are considered indications for a diagnostic test according to current Danish guidelines—e.g., information on previous birth of a child ≥ 4500 g, GDM in a previous pregnancy, familiar predisposition to diabetes or occurrence of glucosuria.

It is unlikely that giving birth in a specific region in itself determines the risk of GDM, and the observed regional differences are more likely explained by an interplay of parameters influencing the definitive GDM prevalence. These include confounding from other unmeasured maternal risk factors, regional differences in the efficacy of risk factor identification, pre-analytic and analytic factors associated with the diagnostic test or accuracy of diagnostic coding. Unfortunately, we were not able to control for these factors.

Further, we do not know how many women in each region or nationally actually underwent diagnostic testing with OGTT for GDM over the study period, and we only have a categorical record of GDM diagnosis (yes/no), without any associated laboratory data from the OGTT.

## Conclusions

The prevalence of GDM increased significantly over time in four of five Danish regions with substantial regional divergence. Up to a 97%, difference in the GDM prevalence was observed between the five Danish regions, which was not explained by available clinical risk factors. This occurred despite national guidelines and raises the question of whether regional variations in screening efficacy, diagnostic procedures or inequality in access to clinical health care may explain the observed differences.

## Supplementary Information

Below is the link to the electronic supplementary material.

Supplementary file1 (PDF 113 kb)

## Data Availability

Due to restrictions in personal data regulations, the register datasets of the current study are generated and analyzed on a closed server hosted by Statistics Denmark and are therefore not publicly available. Access to the Danish National Registries can be provided by applying to Statistics Denmark.
