# Supplementary material for: Regional divergence and time trends in the prevalence of gestational diabetes mellitus: a national Danish cohort study
Source: Acta Diabetol. 2022 Dec 20;60(3):379–86. doi: 10.1007/s00592-022-02013-8 (PMC9931790; doi:10.1007/s00592-022-02013-8)
Supplement: Supplementary file 1 — Supplementary file1 (PDF 113 kb) [file 592_2022_2013_MOESM1_ESM.pdf]

Online Resource Tables 1, 2 and 3 for the article "Regional divergence in the prevalence of gestational diabetes mellitus is not explained by differences in clinical risk factors – a national Danish cohort study"

by Cathrine M. Scheuer, Maria H. Andersen, Elisabeth R. Mathiesen, Lene Ringholm, Clara L. Müller, Jun-Mei Truong, Michelle M. Lie-Olesen, Martin Overgaard, H. David McIntyre, Dorte M. Jensen, Peter Damm, Tine D. Clausen
in **ACTA Diabetologica**

Corresponding author: Cathrine Scheuer
cathrine.munk.scheuer.01@regionh.dk

Online resource Table 1 Detailed classification of explanatory and outcome variables

Variable (code)			
Danish diagnosis coding for gestational diabetes mellitus	Pregnancy, childbirth or maternity leave with gestational diabetes (DO244)<br>Childbirth with gestational diabetes (DO244B)<br>Maternity leave with gestational diabetes (DO244C)<br>Pregnancy with gestational diabetes (DO244D)<br>Pregnancy with insulin-treated gestational diabetes (DO244E)	Prescription classification for assisted reproduction	Gonadotropin and other ovulation stimulating medicaments (MG03G)<br>Gonadotropins (MG03GA)<br>Chorionic gonadotropin (MG03GA01)<br>Gonadotropin, human menopausal (MG03GA02)<br>Serum gonadotropin (MG03GA03)<br>Urofollitropin (MG03GA04)<br>Follitropin alpha (MG03GA05)<br>Follitropin beta (MG03GA06)<br>Lutropin alpha (MG03GA07)<br>Chorionic gonadotropin alpha (MG03GA08)<br>Corifollitropin (MG03GA09)<br>Combinations (MG03GA30)<br>Ovulation stimulating medicaments, synthetic (MG03GB)<br>Cyclofenil (MG03GB01)<br>Clomiphene (MG03GB02)<br>Epimestrol (MG03GB03)
Marital status	Married: Married (G) or Partnership (P)<br>Not married: Widow/widower (E), Divorced (F), Terminated partnership (O), Death (D), Longest living of two partners (L), or Non-married (U)		
Country of origin	Danish country of origin (5100)<br>Unknown country of origin (5001, 5800, and 5999)<br>Non-Danish country of origin (all other codes)		
High educational level	Education below Bachelor's degree:<br>Primary school (10), Lower secondary school (15), Higher examination programmes (20), Vocational education (30), Entry qualification (35), Short-cycle higher education (40), or Medium-cycle higher education (50)<br>High educational level ≥ Bachelor's degree:<br>Bachelor's degree (60), Long cycle higher (70), or Research education (80)		

Online Resource Table 2 GDM prevalence and clinical risk factors for each obstetric department compared to the rest of Denmark, 2017

	No. of births (N)	GDM prevalence (% and N)	Age at delivery (years)	Pre-pregnancy BMI (kg/m²) [a]	High educational level [b]	Married	Nulliparous	Non-Danish country of origin	Assisted reproduction
**Denmark (all hospitals)**	60006	4.2% (2506)	30.3 (± 5.0)	23.3 (18.0 – 38.1)	24.8% (14594)	45.8% (27433)	49.2% (29475)	22.5% (13460)	9.2% (5495)
**Capital Region**									
Rigshospitalet	5438	**3.1% (167)**	**32.1 (± 4.7)**	**22.1 (17.9 – 35.6)**	**50.1% (2676)**	**43.1% (2332)**	**57.4% (3123)**	22.7% (1228)	**11.7% (636)**
Herlev	5373	**3.1% (169)**	**31.1 (± 4.9)**	**22.8 (18.0 – 36.2)**	**37.2% (1954)**	**50.4% (2703)**	**53.2% (2861)**	**32.7% (1755)**	9.7% (522)
Hillerød	4011	**3.5% (141)**	**31.1 (± 5.2)**	**23.1 (17.8 – 36.6)**	**29.1% (1143)**	**54.7% (2191)**	**44.3% (1775)**	**25.3% (1015)**	9.6% (385)
Hvidovre	6760	**2.4% (164)**	**31.1 (± 4.8)**	**22.7 (17.9 – 35.8)**	**34.7% (2283)**	**47.0% (3172)**	**53.9% (3645)**	**31.1% (2101)**	9.5% (642)
Bornholm	256	2.7% (7)	**29.6 (± 5.1)**	**24.2 (18.6 – 38.4)**	**10.4% (26)**	**35.9% (92)**	47.3% (121)	**14.8% (38)**	**3.9% (10)**
**Zealand Region**									
Holbæk	1468	**2.7% (40)**	**29.2 (± 5.3)**	**24.3 (18.1 – 39.8)**	**10.4% (151)**	**39.6% (580)**	**46.5% (681)**	**17.0% (249)**	**6.5% (95)**
Roskilde	2518	**7.2% (182)**	**30.1 (± 5.1)**	**23.6 (18.0 – 39.2)**	**19.0% (470)**	**49.7% (1251)**	**46.9% (1182)**	23.4% (588)	8.4% (211)
Nykøbing Falster	724	**0.8% (6)**	**28.7 (± 5.2)**	**24.5 (17.7 – 38.8)**	**6.3% (45)**	**32.1% (232)**	47.0% (340)	**15.4% (111)**	**3.7% (27)**
Næstved	1836	**5.7% (104)**	**29.0 (± 5.3)**	**24.3 (17.7 – 41.4)**	**8.3% (149)**	**39.4% (723)**	**46.8% (860)**	21.8% (401)	**5.2% (96)**
**Southern Region**									
Odense	4630	**7.1% (329)**	**29.9 (± 5.0)**	**24.0 (18.2 – 39.8)**	**18.3% (834)**	**43.5% (2012)**	**46.3% (2144)**	**20.6% (952)**	**8.2% (378)**
Esbjerg	1889	**5.7% (108)**	**29.3 (± 4.7)**	**24.2 (18.1 – 40.3)**	**11.0% (204)**	**42.4% (801)**	**42.9% (810)**	**18.1% (342)**	**6.4% (120)**
Kolding	3270	**5.4% (174)**	**29.7 (4.9)**	**23.7 (18.0 – 38.9)**	**16.0% (515)**	45.5% (1488)	**44.5% (1454)**	29.1% (716)	8.4% (275)
Aabenraa	1785	**3.0% (54)**	**29.4 (4.9)**	**24.1 (18.0 – 39.8)**	**11.0% (191)**	**43.1% (764)**	**41.4% (739)**	21.9% (388)	**7.2% (128)**
**Central Region**									
Horsens	2160	3.8% (81)	30.2 (± 4.8)	**23.7 (18.3 – 38.7)**	**18.1% (385)**	**48.9% (1056)**	**42.6% (919)**	**16.9% (365)**	**11.6% (251)**
Skejby	4715	4.1% (195)	**30.6 (± 4.7)**	**22.6 (17.9 – 35.8)**	**35.6% (1653)**	**44.1% (2077)**	**53.4% (5511)**	**20.6% (969)**	**11.4% (536)**
Herning-Holstebro	3111	**5.0% (155)**	**29.6 (± 4.7)**	**23.9 (18.0 – 39.4)**	**11.0% (336)**	**50.4% (1567)**	**43.6% (1353)**	**20.2% (629)**	8.9% (277)
Viborg	2237	**2.4% (54)**	**29.9 (± 4.8)**	**23.7 (18.0 – 39.3)**	**16.1% (354)**	45.7% (1022)	**40.9% (916)**	**15.1% (338)**	9.6% (214)
Randers	2220	3.5% (78)	**29.9 (± 5.0)**	**23.8 (18.1 – 38.4)**	**14.1% (307)**	**43.6% (966)**	**44.0% (965)**	**16.8% (373)**	9.9% (219)
**Northern Region**									
Aalborg	3467	**6.5% (226)**	**29.7 (± 4.8)**	**23.8 (17.9 – 39.9)**	**20.9% (710)**	**43.7% (1515)**	50.1% (1734)	**15.7% (543)**	8.6% (298)
Nykøbing-Thisted	596	3.9 % (23)	**29.3 (± 5.0)**	**24.5 (17.9 – 37.2)**	**8.6% (50)**	44.3% (264)	**59.3 % (352)**	**16.1 % (96)**	8.6 % (51)
Vendsyssel	1361	3.2 % (43)	**29.5 (± 4.9)**	**24.2 (17.9 – 39.9)**	**8.3 % (110)**	**40.8 % (555)**	**63.1 % (857)**	**16.0 % (217)**	**7.5 % (102)**

Data for the obstetric departments are reported as distributed in 2022. GDM: Gestational diabetes mellitus. BMI: Body mass index. [b] High educational level: ≥ 12 years' schooling. Categorical data are given as % (N). Continuous data are given as mean (± SD) or [a] median (2.5 - 97.5 percentiles) if data were not normally distributed. Values marked bold were significantly different from the rest of Denmark (*P* values < 0.05).

Online Resource Table 3 Crude and adjusted odds ratios for GDM in all Danish obstetric departments, 2017, with Hillerød as reference

	**Crude odds ratios**		**Model 1 [a]**		**Model 2 [b]**		**Model 3 [c]**	
	OR	95 % CI	OR	95 % CI	OR	95 % CI	OR	95 % CI
**Capital Region**								
Rigshospitalet	0.87	0.69 – 1.09	0.82	0.65 – 1.03	0.89	0.70 – 1.13	0.93	0.73 – 1.18
Herlev	0.89	0.71 – 1.12	0.90	0.72 – 1.13	0.91	0.72 – 1.15	0.84	0.67 – 1.07
Hillerød	Reference		Reference		Reference		Reference	
Hvidovre	**0.68**	**0.54 – 0.86**	**0.69**	**0.55 – 0.86**	**0.70**	**0.55 – 0.88**	**0.65**	**0.51 – 0.82**
Bornholm	0.77	0.36 – 1.67	0.86	0.40 – 1.86	0.68	0.27 – 1.69	0.73	0.29 – 1.83
**Zealand Region**								
Holbæk	0.77	0.54 – 1.10	0.88	0.61 – 1.26	**0.66**	**0.46 – 0.96**	**0.67**	**0.46 – 0.98**
Roskilde	**2.14**	**1.71 – 2.68**	**2.32**	**1.85 – 2.91**	**2.10**	**1.65 – 2.67**	**2.13**	**1.67 – 2.71**
Næstved	**1.65**	**1.27 – 2.14**	**1.93**	**1.49 – 2.50**	**1.49**	**1.14 – 1.95**	**1.54**	**1.17 – 2.02**
Nykøbing Falster	**0.23**	**0.10 – .52**	**0.27**	**0.12 – 0.62**	**0.18**	**0.07 – 0.44**	**0.19**	**0.08 – 0.47**
**Southern Region**								
Odense	**2.10**	**1.71 – 2.57**	**2.32**	**1.90 – 2.84**	**1.93**	**1.57 – 2.38**	**2.00**	**1.59 – 2.43**
Esbjerg	**1.66**	**1.29 – 2.15**	**1.93**	**1.49 – 2.49**	**1.53**	**1.17 – 1.99**	**1.61**	**1.22 – 2.11**
Kolding	**1.55**	**1.24 – 1.95**	**1.73**	**1.38 – 2.18**	**1.54**	**1.22 – 1.94**	**1.54**	**1.22 – 1.96**
Aabenraa	0.86	0.62 – 1.18	0.98	0.71 – 1.35	0.76	0.55 – 1.06	0.80	0.58 – 1.12
**Central Region**								
Horsens	1.07	0.81 – 1.41	1.16	0.88 – 1.53	1.01	0.76 – 1.35	1.05	0.79 – 1.41
Skejby	1.18	0.95 – 1.48	**1.25**	**1.00 – 1.56**	**1.31**	**1.05 – 1.64**	**1.32**	**1.05 – 1.66**
Herning-Holstebro	**1.44**	**1.14 – 1.82**	**1.63**	**1.29 – 2.06**	**1.36**	**1.07 – 1.73**	**1.41**	**1.11 – 1.80**
Viborg	**0.68**	**0.49 – 0.93**	0.75	0.55 – 1.03	**0.62**	**0.45 – 0.85**	**0.68**	**0.49 – 0.95**
Randers	1.00	0.75 – 1.32	1.10	0.83 – 1.46	0.94	0.70 – 1.26	1.02	0.76 – 1.37
**Northern Region**								
Aalborg	**1.91**	**1.54 – 2.37**	**2.15**	**1.73 – 2.67**	**1.83**	**1.46 – 2.28**	**1.92**	**1.53 – 2.41**
Nykøbing-Thisted	0.90	0.63 – 1.27	1.26	0.81 – 1.99	1.08	0.69 – 1.71	1.11	0.70 – 1.76
Vendsyssel	1.10	0.70 – 1.73	1.01	0.72 – 1.44	0.83	0.58 – 1.78	0.78	0.55 – 1.14

Data for the obstetric departments are reported as distributed in 2017. GDM: Gestational diabetes mellitus. OR: Odds ratio. CI: Confidence interval.
[a] Model 1 adjusted for age at delivery.
[b] Model 2 adjusted for model 1 and pre-pregnancy BMI.
[c] Model 3 adjusted for model 2 and educational level, marital status, parity, country of origin and assisted reproduction.
